# Balanced Shh signaling is required for proper formation and maintenance of dorsal telencephalic midline structures

**DOI:** 10.1186/1471-213X-10-118

**Published:** 2010-11-29

**Authors:** Diana S Himmelstein, Chunming Bi, Brian S Clark, Brian Bai, Jhumku D Kohtz

**Affiliations:** 1 Developmental Biology and Department of Pediatrics, Children's Memorial Research Center and Feinberg School of Medicine, Northwestern University, Chicago, IL, USA; 2 Department of Cell Biology, Neurobiology and Anatomy, Medical College of Wisconsin, Milwaukee, WI, USA; 3 Department of Genetics, Case Western Reserve University, Cleveland, OH, USA

## Abstract

**Background:**

The rostral telencephalic dorsal midline is an organizing center critical for the formation of the future cortex and hippocampus. While the intersection of WNTs, BMPs, and FGFs establishes boundaries within this critical center, a direct role of Shh signaling in this region remains controversial. In this paper we show that both increased and decreased Shh signaling directly affects boundary formation within the telencephalic dorsal midline.

**Results:**

Viral over-expression of Shh in the embryonic telencephalon prevents formation of the cortical hem and choroid plexus, while expanding the roof plate. In a transgenic model where cholesterol-lacking ShhN is expressed from one allele (*ShhN/+*), genes expressed in all three domains, cortical hem, choroid plexus and roof plate expand. In *Gli1/2 -/- *mutant brains, where Shh signaling is reduced, the roof plate expands, again at the expense of cortical hem and plexus. Cell autonomous activation of Shh signaling in the dorsal midline through Gdf7-driven activated Smoothened expression results in expansion of the *Wnt3a*-expressing cortical hem into the plexus domain. In addition, developmental stage determines dorsal midline responsiveness to Shh.

**Conclusions:**

Together, these data demonstrate that balanced Shh signaling is critical for maintaining regional boundaries within the dorsal midline telencephalic organizing center.

## Background

The telencephalic dorsal midline contains two organizing centers: the roof plate and cortical hem [1,2]. The roof plate is initially induced by signals from the overlying epidermal ectoderm, and once established, provides a secondary source of secreted TGFβ-family members along the entire dorsal midline of the developing neural tube [3]. Fate-mapping experiments show that the roof plate is derived from *Wnt1*-expressing cells in the overlying neuroectoderm, and develops from lineage-restricted cellular compartments [4]. Evidence that the roof plate may be an organizer stems from genetic ablation experiments demonstrating roof plate-dependent dorsal interneuron specification in the spinal cord, and cortical and choroid plexus development in the telencephalon [5-7]. In addition, it has been shown that the roof plate directs choroid plexus formation through a cell non-autonomous mechanism [5]. The cortical hem, originally identified as an embryonic structure marked by *Wnt *expression [8], exhibits hippocampal organizer activity [9]. Targeted inactivation of *Wnt3a *and the signaling co-factor *Lef-1 *confirm the hem's role in hippocampal growth and development [10,11].

The intersection of multiple secreted factors expressed in the telencephalic midline are known to contribute to the patterning of this region. These include BMPs, WNTs and FGFs [12-14]. FGF signals from the rostral forebrain regulate anterior-posterior regionalization in the telencephalon [15]. The current model suggests that a number of FGFs, specifically FGF8, function to coordinate the competing morphogenic signals expressed from the dorsal and ventral midlines [16].

However, recent questions have been raised as to a direct role of the secreted signaling protein Shh in patterning the dorsal telencephalic midline. While Shh is expressed in the ventral telencephalic midline as early as E10.5, a time when the first invagination of the dorsal midline region occurs, it is not detectable in the dorsal telencephalic midline. However, disruption of Shh signaling through mutation of *Shh *or its downstream targets affects dorsal midline patterning in both humans and mice [17-19]. Genetic interactions between *Shh *and *Gli3 *suggested a mechanism [20] whereby Shh expression in the telencephalic ventral midline represses the transcriptional repressor Gli3 through an activity gradient [21]. However, loss of both *Shh *copies in *Gli3 *mutants fails to rescue telencephalic dorsal midline defects, challenging the idea that Shh acts solely through a Gli3-dependent mechanism [22]. Together these data suggest that the role of Shh in dorsal telencephalic midline patterning is still not well understood.

In this paper we show that Shh expression from the dorsal extent of the zona limitans intrathalamica (ZLI, [23]) is positioned correctly to directly influence gene expression boundaries critical for telencephalic dorsal midline formation, specifically the choroid plexus and cortical hem. Using mouse models that contain increased or decreased Shh activity in the developing forebrain, we show that balanced Shh signaling is required for proper telencephalic dorsal midline development. Further, we show that disruption of FGF signaling does not result in dorsal midline patterning defects resembling the Shh loss- or gain-of-function mouse models, suggesting that Shh signaling in the dorsal midline is not mediated by FGFs. Together, these data support a direct role of Shh in patterning the dorsal telencephalic midline.

## Results

### *Shh *is expressed adjacent to the *Lhx5*-expressing roof plate domain in the embryonic telencephalon

**Figure 1:**
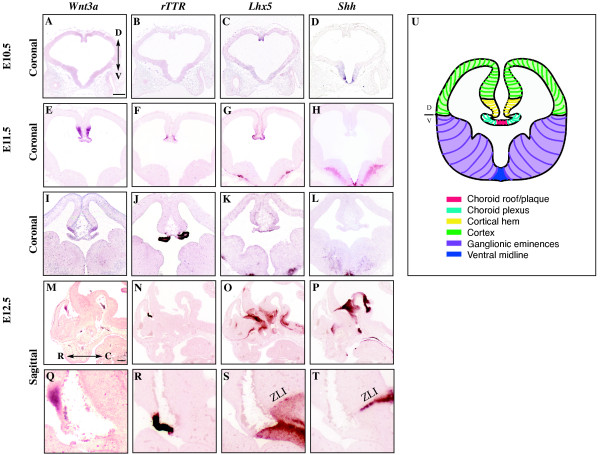
**Relationship of Shh and dorsal telencephalic midline markers at E10.5, E11.5, and E12.5**. RNA in situ hybridization analysis of dorsal midline telencephalic genes, *Wnt3a*, *rTTR*, *Lhx5*, and ventral midline expression of *Shh*. Coronal sections [A-L], sagittal sections (M-T). E10.5 (A-D), E11.5 (E-H), and E12.5 (I-T). Scale bars = 200 μm. U is a schematic depicting the regional boundaries corresponding to the observed gene expression domains in a coronal section at mouse embryonic day 12.5. D, Dorsal, V, Ventral, R, Rostral, C, Caudal.

At E12.5, the telencephalic dorsal midline is delineated by three regions based on morphology and gene expression [8]: the *Wnt3a*-expressing cortical hem, the *rTTR*-expressing choroid plexus, and the *Lhx5*-expressing roof plate (Figure 1). At E10.5, prior to the appearance of *rTTR *in the choroid plexus, *Wnt3a *and *Lhx5 *overlap in the dorsal midline (Figure 1A, C). Between E11.5 and E12.5, the appearance of the choroid plexus divides the roof and hem boundaries from each other (Figure 1E-G, I-K). In E10.5-E12.5 coronal sections, *Shh *expression is restricted to telencephalic ventral midline, and therefore unlikely to directly influence formation of dorsal telencephalic midline structures (Figure 1D, H, L). However, sagittal analyses at these stages show that dorsal diencephalic extension of *Shh *in the ZLI is adjacent to the *Lhx5*-expressing telencephalic roof plate, the region known to be a telencephalic dorsal midline organizer (Figure 1O, P, S, T). The latter raises the possibility that Shh signaling directly influences dorsal midline structures/boundaries during the time that the choroid plexus becomes juxtaposed between the cortical hem and roof plate.

### Increased Shh signaling changes telencephalic dorsal midline morphology and boundaries

**Figure 2:**
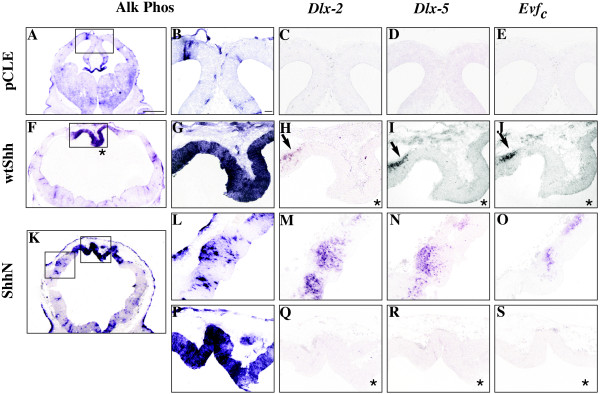
**Shh activation ventralizes the dorsal lateral telencephalon, but not dorsal midline**. E9.5 mouse brains were infected with wtShh, ShhN or pCLE (alkaline phosphatase alone) expressing viruses, and harvested at E12.5. Alkaline phosphatase visualization of viral infections (A, B, F, G, K, L, P) and adjacent section RNA in situ hybridization analysis of the dorsal midline and lateral E12.5 telencephalon of virally infected brains: *Dlx-2 *(C, H, M, Q), *Dlx*-5 (D, I, N, R), *Evf_c _*(E, J, O, S). n = 3. Arrows indicate ventral gene expression in the cortex; asterisks identify the dorsal midline. Scale bar in A represents 1 mm and panels A, F and K are photographed at the same magnification (1x). Scale bar in B represents 65 μm and panels B-E, G-J, and L-S are photographed at the same magnification (10×).

**Figure 3:**
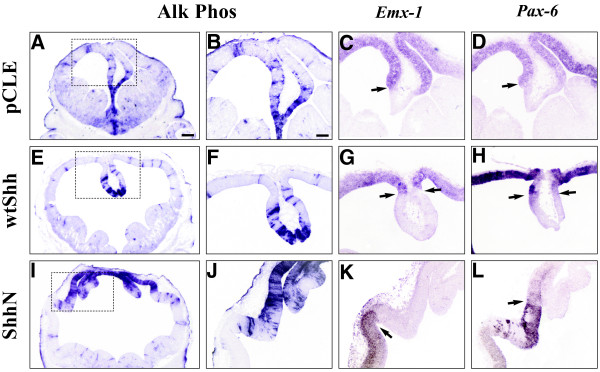
**Shh activation shifts cortical gene expression laterally**. E9.5 mouse brains were infected with wtShh, ShhN or pCLE (alkaline phosphatase alone) expressing viruses, and harvested at E12.5. Alkaline phosphatase visualization of viral infections (A, B, E, F, I, J) and adjacent section RNA in situ hybridization analysis of E12.5 virally infected brains, *Emx-1*: C, G, K, *Pax-6*: D, H, L (arrows indicate shifted borders of *Emx-1 *and *Pax-6 *expression). Control pCLE (A-D), wtShh (E-H), ShhN [I-L]. n = 3. All panels in this figure were photographed at the same magnification (5x). Scale bar in A represents 45 μm and panels A, E and I are shown at the same magnification. Scale bar in B represents 130 μm and panels B-D, F-H, and J-L are shown at the same magnification.

**Figure 4:**
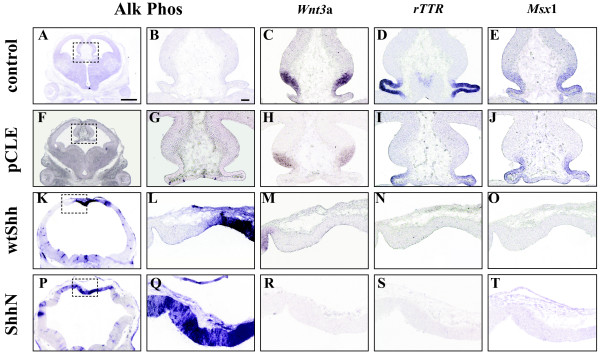
**Shh activation prevents cortical hem and choroid plexus formation**. E9.5 mouse brains were infected with wtShh, ShhN, and pCLE (alkaline phosphatase alone) expressing viruses, or uninfected littermate control (control), and harvested at E12.5. Alkaline phosphatase visualization of viral infections (A, B, F, G, K, L, P, Q) and adjacent section RNA in situ hybridization analysis of the dorsal midline of E12.5 virally infected brains, *Wnt3a *(cortical hem): C, H, M, R. *rTTR *(choroid plexus): D, I, N, S, *Msx1 *(cortical hem + plexus): E, J, O, T. n = 3. Scale bar in A represents 1 mm and panels A, F, K and P are photographed at the same magnification (1x). Scale bar in B represents 65 μm and panels B-E, G-J, L-O and Q-T are photographed at the same magnification (10×).

**Figure 5:**
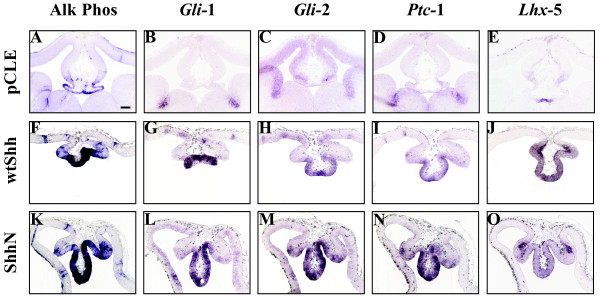
**The dorsal midline responds to Shh activation by expanding the *Lhx5*- expressing roof plate domain**. E9.5 mouse brains were infected with wtShh or ShhN expressing viruses, or pCLE (alkaline phosphatase alone), and harvested at E12.5. Alkaline phosphatase visualization of viral infections (A, F, K) and adjacent section RNA in situ hybridization analysis of the dorsal midline of E12.5 virally infected brains, *Gli-1*: B, G, L, *Gli-2*: C, H, M, *Ptc-1*: D, I, N, *Lhx-5 *(roof plate): E, J, O. n = 3. Scale bar in A represents 130 μm and all panels in this figure are photographed at the same magnification (5×).

In order to directly test the influence of Shh on dorsal midline development, we used ultrasound image-guided injections to introduce viruses expressing cholesterol-lacking Shh (ShhN) and full length Shh (wtShh) into the E9.5 embryonic forebrain ventricle. This technique provides a powerful approach to disentangle the spatial and temporal roles of Shh in brain development. At E12.5, three days after viral misexpression, wtShh and ShhN virally infected brains are characterized by expanded lateral ventricles and a thinned cortex. The dorsal midline fails to properly invaginate and cortical hem and choroids plexus differentiation is disrupted (Figure 2F, K, 3E, I, 4K, P, 5F, K).

Previous experiments showed that ShhN in vitro and wtShh in vivo ventralizes the dorsal telencephalon by activating Dlxs and Evfs at embryonic ages [24-28]. Characterization of the E12.5 telencephalon from wtShh and ShhN viral infections performed at E9.5 confirms that cortical tissue is ventralized, expressing *Dlx2 *(Figure 2H, M), *Dlx5 *(Figure 2I, N) and *Evf_c _*(Figure 2J, O). However, the dorsal midline is refractory to ventralization by ectopic Shh expression (Figure 2Q-S). While the dorsal midline is not ventralized by ectopic Shh activation, cortical genes *Emx-1 *and *Pax-6 *are shifted laterally (*Emx-1*, Figure 3G, K; *Pax-6*, Figure 3H, L), showing that the dorsal midline has not been transformed to cortical or striatal fate.

Since dorsal midline morphology is significantly altered by Shh activation, it is likely that the dorsal midline is still influenced by Shh signaling. Therefore, we next addressed how genes distinguishing the cortical hem, choroid plexus and roof plate may be altered by Shh activation. Figure 4 shows that Shh activation results in the loss of hem (*Wnt3a*, Figure 4M, R) and plexus (*rTTR*, Figure 4N, S) structures. *Msx-1*, which is normally expressed by both hem and plexus, is also lost upon Shh activation (Figure 4O, T). Therefore, Shh activation results in the loss of specific midline structures, but does not transform the dorsal midline to a ventral fate.

We next asked if Shh activates downstream targets in the dorsal midline, or whether the remaining dorsal midline tissue is refractory to Shh signaling. Figure 5 shows that the dorsal midline responds to Shh activation by ectopic activation of its transcriptional target *Gli-1 *[29] (Figure 5G, L), its co-receptor *Ptc-1 *[30-32] (Figure 5I, N), and expansion of the roof plate domain marked by *Lhx5 *expression (Figure 5J, O). Therefore, Shh activation causes roof plate expansion at the expense of choroid plexus and cortical hem.

**Figure 6:**
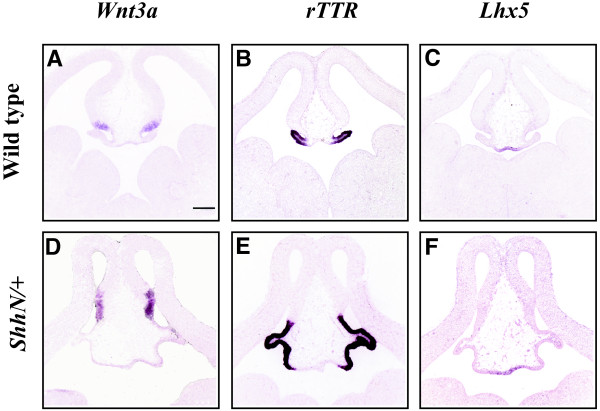
**Activated ShhN from the endogenous Shh source expands dorsal midline telencephalic cortical hem, plexus and roof plate**. RNA in situ hybridization analysis of E12.5 wild type littermate control (A-C) and *ShhN/+ *(D-F) telencephalon. *Wnt3a*: A, D, *rTTR*: B, E, *Lhx5*: C, F. n = 3. Scale bar in A represents 200 μm and all panels in this figure are photographed at the same magnification (5×).

The experiments in Figure 2, 3, 4, and 5 show the effects of ectopic Shh activation by viral delivery to E9.5 telencephalic dorsal midline. In order to address if increased Shh signaling from its normal source (ventral midline or ZLI) would affect telencephalic dorsal midline formation, we utilized an activated Shh transgenic model where one allele of Shh is replaced by ShhN (*ShhN/+*) [33]. In this model, ShhN is proposed to travel further, resulting in Shh activation in the dorsal telencephalon. Figure 6 shows that all three telencephalic dorsal midline boundaries are expanded in *ShhN/+ *mice (Figure 6D-F). While viral activation and genetic modification both cause roof plate *Lhx5 *expansion, *rTTR *and *Wnt3a *expand in *ShhN/+ *mice (Figure 6) and are absent in brains injected with Shh virus (Figure 4). Together, these data not only support the idea that increased Shh signaling alters telencephalic dorsal midline patterning, but that timing and dose are critical factors in determining the outcome.

### Telencephalic dorsal midline defects result from decreased Shh signaling

**Figure 7:**
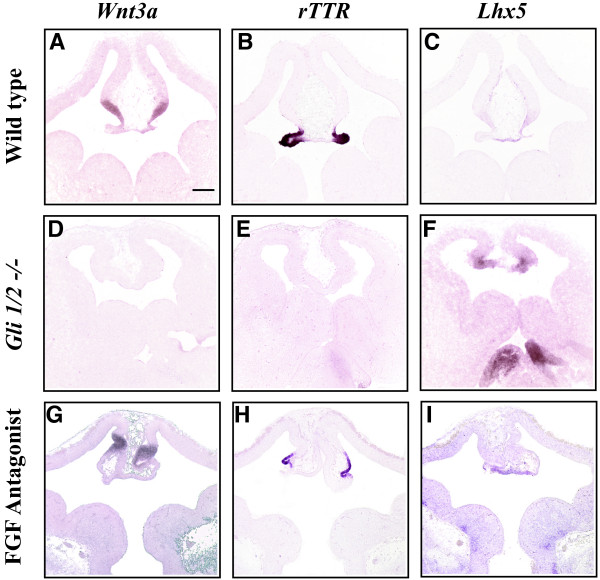
**Loss of cortical hem and plexus, and expanded roof plate in *Gli1/2 -/- *brains differ from dorsal midline defects resulting from FGF inhibition**. RNA in situ hybridization analysis of E12.5 wild type littermate control (A-C), *Gli 1/2 -/- *double knock-out (D-F), and FGF antagonist-injected (G-I) telencephalon. *Wnt3a*: A, D, G, *rTTR*: B, E, H, *Lhx5*: C, F, I. n = 2. Scale bar in A represents 200 μm and all panels in this figure are photographed at the same magnification (5×).

Increased Shh signaling, both indirectly through loss of a repressor (Gli3, [8]) and directly through transgenic or viral expression (this paper) can affect telencephalic dorsal midline boundaries. However, it is unknown whether Shh normally plays a direct role in patterning the telencephalic dorsal midline. The dramatic loss of both dorsal and ventral telencephalic midline structures in *Shh -/- *mutants supports the idea that Shh is critical for proper formation of both regions [34]. Since Shh has been shown to activate transcriptional targets through the action of Gli1 and Gli2, combined loss of these two transcription factors reduces, but does not eliminate direct Shh signaling [35,36]. Figure 7 shows that reduced Shh signaling in *Gli1/2 -/- *mutants results in the loss of *Wnt3a *(Figure 7D) and *rTTR *(Figure 7E) in the hem and plexus, respectively. In place of the hem and plexus, the *Gli1/2 -/- *mutant telencephalic dorsal midline now expresses *Lhx5 *throughout (Figure 7F), without proper thinning to a single epithelial layer characteristic of the roof plate [1]. This suggests that Shh signaling is required for inducing proper morphology and gene expression boundaries that establish the hem, plexus and roof relationships.

The current model predicts that Shh indirectly influences dorsal midline development through the action of FGF8 [18,33]. Shh expression from the ventral midline is necessary for the maintenance of FGF8 expression [37], and FGF8 is thought to restrict the spread of BMPs and WNTs in the dorsal telencephalon [12]. Given the hypothesis that Shh acts through FGF8 to influence the dorsal midline, we investigated the effects of FGF inhibition during dorsal telencephalic midline patterning. An FGF receptor tyrosine kinase inhibitor (PD173074, Calbiochem) was injected into mouse E9.5 forebrain, and gene expression changes were examined at E12.5. While the FGF inhibitor interferes with the morphological development of the dorsal midline, *Wnt3A *and *rTTR *are still expressed in the cortical hem and choroid plexus structures, respectively (Figure 7G, H). In addition, *Lhx5 *expansion and thickening of the roof plate is not observed (Figure 7I). Therefore, the dorsal midline defects resulting from reduced Shh signaling (*Gli1/2 -/-*) are distinct from reduced FGF signaling, raising the possibility that Shh signaling in the telencephalic dorsal midline may be independent of FGF. However, we cannot rule out the possibility that FGF inhibition is incomplete, and then fails to generate the complete roof plate phenotype. Future experiments that include both gain and loss of function FGF action in the telencephalon combined with Shh activation will be necessary to determine whether Shh acts through FGF in this region.

### Cell autonomous increase in telencephalic dorsal midline Shh signaling alters cortical hem/plexus boundary

**Figure 8:**
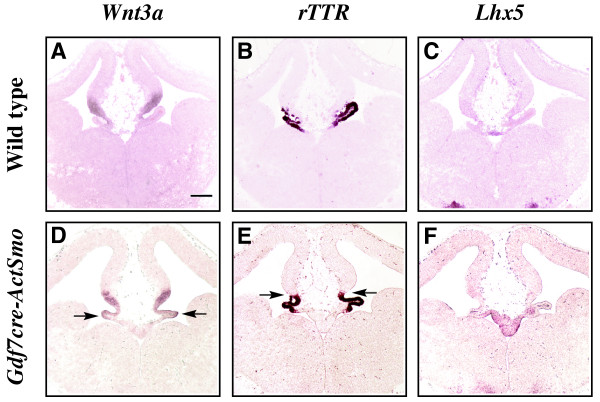
**Cell autonomous Shh-activated roof plate expansion causes *Wnt3a *expansion into choroid plexus domain**. RNA in situ hybridization analysis of E12.5 wild type littermate control (A-C) and activated Smoothened expressed in the roof plate through *GDF7cre *regulation (D-F). *Wnt3a*: A, D, *rTTR*: B, E, *Lhx5*: C, F. n = 3. Arrows point to *Wnt3a *expansion (D) and *rTTR *expansion (E). Scale bar in A represents 200 μm and all panels in this figure are photographed at the same magnification (5×).

In order to distinguish between cell-autonomous and non-autonomous Shh activity, we next tested the effects of *Gdf7*-promoted activated Smoothened on telencephalic dorsal midline gene expression boundaries and morphology. Using a *Gdf7cre *transgenic mouse [6] crossed to a conditionally activated Smoothened (*ActSmo*) [38], it is possible to limit Shh activation to roof plate cells, and to a small group of hem and plexus cells. Figure 8 shows that cell autonomous activation of Shh through *act-Smo *expression in the Gdf7 domain increases roof plate thickness, and prevents formation of the single epithelial layer characteristic of the roof plate [1]. *Wnt3a*, which is normally limited to the cortical hem, expands into the choroid plexus region (arrows, Figure 8D). In addition, *rTTR *expression expands slightly dorsally into the cortical hem region (arrows, Figure 8E), and the choroid plexus appears somewhat reduced in size. Together these data suggest that Shh-dependent cell autonomous changes in the roof plate shifts boundaries between hem and plexus.

### Time-dependent effects of Shh ectopic activation on dorsal midline boundaries

**Figure 9:**
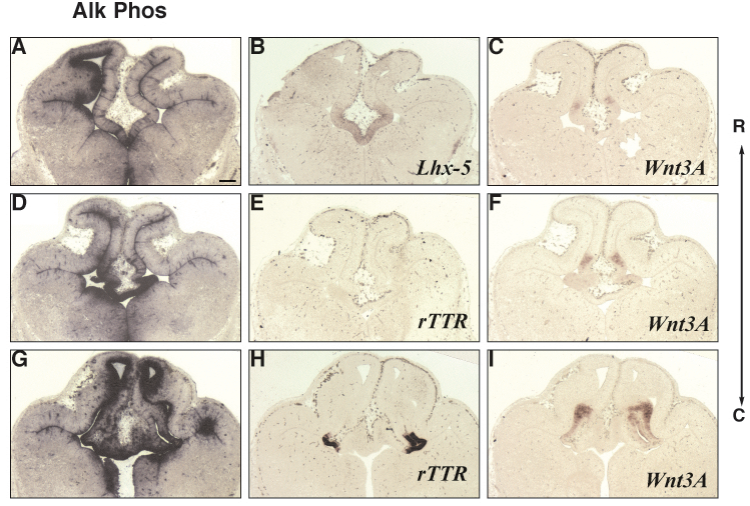
**Shh expands the roof plate, but does not prevent formation of the hem in E10.5 injected telencephalon**. E10.5 mouse telencephalon was injected with wtShh expressing retrovirus, and harvested at E12.5. Coronal sections were stained for virally infected cells using an alkaline phosphatase substrate at different rostral caudal positions (A, D, G). RNA in situ hybridization analysis was performed on adjacent sections; sections were probed using the following: *Lhx5 *(B), *Wnt3A *(C, F, I) and *rTTR *(E, H). n = 3. Uninjected littermate controls were identical to those shown in Figures 6, 7 and 8. Scale bar in A represents 130 μm and all panels in this figure are photographed at the same magnification (5×). R, Rostral, C, Caudal.

The appearance of the telencephalic choroid plexus (between E11.5 and E12.5) follows the formation of the roof plate and cortical hem (between E9.5 and E11.5). The appearance of *Shh *in the ZLI occurs between E10.5 and E12.5 [39]. If balanced Shh signaling were required to maintain relationships between the telencephalic roof plate, hem, and plexus, then it would be expected that the roof plate remains competent to respond to Shh signaling later than E9.5. Therefore, we next asked whether increased Shh signaling affects dorsal midline patterning after roof plate and hem formation have already begun. Figure 9 shows E12.5 dorsal telencephalic midline analysis of brains infected with wtShh virus at E10.5. Roof plate expansion, as indicated by *Lhx5 *expression, is still observed (Figure 9B). In addition, the choroid plexus gene *rTTR *is not detected rostrally in the telencephalon (Figure 9E), but is detected in more caudal sections at the level of the diencephalon (Figure 9H). The morphological invagination of the choroid plexus epithelium is apparent without induction of *rTTR*. The cortical hem can be distinguished both morphologically and by expression of *Wnt3A *(Figure 9C, F, I). Expansion of the hem into the plexus domain is also observed (Figure 9F, I), similar to that found in *GDF7cre-ActSmo *brains (Figure 8D). Therefore, hem and plexus structures change in Shh responsiveness between E9.5 and E10.5, while the roof plate retains its earlier sensitivity. The latter is consistent with the idea that Shh is important for maintaining the roof plate after its formation.

## Discussion

In this paper, we show that increased or decreased Shh signaling disrupts dorsal telencephalic midline development, suggesting that balanced Shh signaling is required for the proper formation and maintenance of the telencephalic dorsal midline. We show that Shh activity can directly influence dorsal midline patterning through ectopic viral expression at E9.5 (Shh^E9.5vir^). The Shh^E9.5vir ^telencephalon lacks both cortical hem and choroid plexus. In addition, the Shh^E9.5vir ^telencephalic dorsal midline is refractory to ventralization, with expanded expression of the roof plate gene *Lhx5*. Recent characterization of the *ShhN/+ *telencephalon also shows *Lhx5 *roof plate expansion accompanied by a loss of cortical hem and choroid plexus [33]. These results are consistent with the finding that ShhN, the cholesterol lacking form of Shh, is more potent in telencephalic explant assays [25], and exhibits an extended range of signaling [33,40,41]. Also consistent with this idea is that ectopic expression of ShhN in the dorsal midline results in a more extreme phenotype than wtShh (Figure 2F vs 2K, Figure 3E vs 3I, Figure 4K vs 4P, Figure 5F vs 5K). While our analysis of Shh^E9.5vir ^using wtShh or ShhN supports that *ShhN/+ *mutant phenotypes in the telencephalon result from increased Shh activity, our analysis of *ShhN/+ *dorsal midline defects differs from that of Huang et al. [33]. Both analyses show that *ShhN/+ *mutants exhibit roof plate expansion. However, unlike Huang et al. [33], our analysis shows that *ShhN/+ *mutants display expanded cortical hem and plexus structures along with the expanded roof plate, generating a larger dorsal midline. Phenotypic differences in *ShhN/+ *may result from differential cre-induced removal of the *ShhN *conditional allele (*EIIAcre *compared with *Sox2Cre*), or possibly from mouse strain differences.

The source of Shh in *ShhN/+ *mutants derives from adjacent or ventral domains. The dorsal midline expansion in *ShhN/+ *embryos may be attributed to increased Shh signaling from the ventral telencephalon, increased signaling from the diencephalic ZLI, or both. In contrast, viral delivery results in direct expression of Shh in the dorsal midline. Thus, the level of exposure at the dorsal midline is likely to be higher in Shh^E9.5vir ^compared with *ShhN/+*. The use of viral delivery methods can help elucidate direct effects of Shh activity on dorsal midline boundary formation. Another important difference between these two methods of ectopic Shh expression is the timing of delivery. In Shh^E9.5vir^, ectopic Shh activity begins at E9.5, after the telencephalon has begun to form. In *ShhN/+*, increased Shh is co-expressed with endogenous Shh concurrent with formation of the telencephalon. These data suggest that the ability of Shh to prevent or expand cortical hem and plexus may be determined by changes in source, level, or timing of exposure to Shh.

In a direct test of the effect of timing of Shh exposure on dorsal midline patterning, Shh viral infections were performed at E10.5 (Shh^virE10.5^). Several interesting differences are detected when dorsal midline patterning is compared in Shh^virE9.5 ^and Shh^virE10.5 ^brains. While the roof plate expands in response to Shh at both delivery time points, opposite effects occur on the hem, which expands after E10.5 Shh exposure rather than diminishing (E9.5). In the Shh^virE10.5 ^telencephalon, morphological appearance of the choroid plexus occurs without activation of the plexus-specific gene *rTTR*, suggesting that choroid plexus development is still sensitive to Shh activity at this time. Differential alteration of dorsal midline patterning as a result of different methods of increased Shh activity supports the idea that the dorsal midline, and particularly the roof plate, is a sensitive responder of Shh signaling.

It has previously been proposed that Shh influences dorsal telencephalic midline patterning through a Gli3 gradient or FGF signaling rather than through direct signaling [21,22,33]. However, the sensitivity of the dorsal telencephalic midline to ectopic Shh signaling suggests that Shh can directly influence patterning of this region. In addition, decreased Shh signaling in mice lacking both *Gli1 *and *Gli2 *results in dorsal telencephalic midline defects similar to that found when Shh is increased (Shh^E9.5vir^): loss of cortical hem and choroid plexus, with a concomitant expansion of the roof plate gene *Lhx5*. Therefore, both increased and decreased Shh signaling can have similar effects on telencephalic dorsal midline patterning.

A shared aspect between *Gli1/2 -/- *mutants, Shh^E9.5vir^, Shh^E10.5vir ^and *ShhN/+ *is *Lhx5*-expressing roof plate expansion. Cell autonomous Shh activation in the roof plate (*GDF7cre-ActSmo*) also results in roof plate and hem expansion with minimal plexus expansion, supporting a direct role of Shh in maintaining roof plate/hem/plexus boundaries. These data further support previous data suggesting a cell-non-autonomous role of the roof plate, presumably mediated by BMPs, influencing choroid plexus formation [5]. Together, these data show that the roof plate is particularly sensitive to Shh signaling, requiring a balanced level of Shh activity for proper morphology and gene expression. Interestingly, mutations in Shh or its downstream targets result in the developmental defect holoprosencephaly [19,42], and interference in roof plate function has been linked to a milder form of the disease affecting dorsal hemispheric separation [43-45]. Thus, our data suggests that Shh-mediated roof plate effects may be responsible for the dorsal midline defects found in holoprosencephaly.

We show that both increases and decreases in Shh signaling ventrally (*ShhN/+ *and *Gli1/2 -/-*) and dorsally (wtShh and ShhN injections) significantly alter dorsal midline gene expression. The current model predicts that Shh acts through FGF8 to influence the telencephalic dorsal midline [18,33]. However, the dorsal midline phenotype obtained with reduced FGF signaling and altered Shh signaling may be distinct. In particular, roof plate expansion of *Lhx5 *is common to both reduced and increased Shh signaling, but absent from reduced FGF signaling. It is possible that the failure to observe common roof plate defects between FGF inhibition and Shh alterations is due to inefficient FGF reduction. However, an argument against this is that the FGF inhibitor increases rather than prevents *Wnt3a *expression in the hem, as observed with altered Shh signaling. If FGF inhibition is inefficient, the phenotype obtained would be expected to be a subset of the complete phenotype rather than an opposing phenotype. Another argument against inefficient FGF inhibition is the appearance of obvious dorsal midline morphological defects in response to the FGF inhibitor. If FGF inhibition were inefficient, gene expression boundaries may be changed without obvious morphological defects. Although further manipulations are necessary to definitively rule out Shh action through FGFs in the dorsal midline, the present data support the idea that Shh acts directly rather than through FGF signaling to maintain boundary relationships in the dorsal telencephalic midline.

Experiments in this paper indicate that the telencephalic dorsal midline, specifically the roof plate, is a direct Shh target. What is the most likely source of Shh that directs telencephalic dorsal midline formation? During the time that mouse telencephalic dorsal midline boundaries are established (E10.5-E12.5), the *Lhx5 *expression domain in the roof plate becomes juxtaposed to Shh expression in the ZLI. This is most apparent at E12.5 (Figure 1S, T). The ZLI is a strip of cells that act as a signaling center to induce expression of regionally restricted transcription factors in flanking areas [23,46]. The ZLI demarcates the boundary between the prethalamus and the functionally distinct thalamus and a gradient of Shh expression from the ZLI is required for establishing regional identity in these bordering structures [47]. While DiI labeling revealed that the *Shh*-expressing ZLI initially forms from cells in the alar plate, both *Shh *expression in the ZLI and subsequent diencephalic development depend on a source of Shh secreted from the basal plate of the forebrain [46]. Taken together with results in this paper, reduced or increased Shh activity resulting from mutations in *Shh *or its downstream targets would be predicted to affect ZLI formation, and ultimately influence patterning and/or maintenance of the telencephalic dorsal midline.

## Conclusions

Our data suggests that the telencephalic roof plate is a sensitive responder to changes in Shh activity, and that its expansion occurs in response to increased or decreased Shh signaling. We show that roof plate expansion in response to changes in Shh signaling occurs at the expense of the hem and plexus or can lead to expansion of the hem and plexus. In addition, responsiveness of the telencephalic dorsal midline to Shh signaling changes between E9.5 and E10.5 in mice. The structural relationship of Shh and dorsal midline markers raises the possibility that dorsally expressed Shh from the diencephalic ZLI is a source of Shh signaling to the telencephalic dorsal midline. We propose that imbalanced Shh signaling directly changes dorsal midline boundaries between the roof plate, choroid plexus and cortical hem.

## Methods

### Animals and Surgery

*ShhN floxed *mice [48] were obtained from Dr. A. McMahon, and crossed to EIIAcre mice (Jackson Lab) to generate *ShhN/+ *embryos at E12.5. *ActSmo floxed *mice [38] were crossed to *GDF7cre *mice [6] to generate *GDF7cre-ActSmo *E12.5 embryos. Animals used in these studies were maintained according to protocols approved by Institutional Animal Care and Use Committee at Children's Memorial Hospital Research Center. Timed-pregnant Swiss Webster mice used for injections were obtained from Taconic breeding laboratories. Embryonic day 0.5 is defined as noon of the day a vaginal plug was found after overnight mating. Detailed animal care, preparation for surgery and the use of the ultrasound scanner (UBM scanner) have been previously described [49].

### Viral expression and embryonic injections

Shh full-length cDNA (wtShh) and the N-terminal fragment (ShhN) were inserted into the pCLE viral vector backbone, and pseudo-typed retrovirus was produced as previously described [26]. Viruses were titered on the C17 neural cell line [50]. Western analysis (not shown) on C17 infected extracts was performed with rabbit anti-Shh antibody (1:5000, R&D). Viruses were diluted to 5 × 10^7 ^cfu/ml in PBS containing 80 μg/ml polybrene (Sigma). FGF receptor tyrosine kinase inhibitor PD173074, Calbiochem) was dissolved in DMSO and diluted to 25 μM in PBS containing 80 μg/ml polybrene (sigma). FGF receptor tyrosine inhibitor PD173074, calbiochem) was dissolved in DMSO and diluted to 25 μM in PBS containing 80 μg/ml polybrene. 1-1.5 μl of virus or FGF inhibitor was injected into the E9.5 or E10.5 mouse telencephalon using ultrasound-guided in utero injection, as previously described [26,49,51]. Embryos were harvested 2 or 3 days after injection, as indicated.

### In situ hybridization

In situ hybridization was performed by modification of Schaeren-Wiemers and Gerfin-Moser [52] as previously described [27], with the exception of the *rTTR *probe, which was visualized using the method of Tekki-Kessaris et al. [53]. The *Lhx5 *in situ probe was made from pGEM-Teasy (Promega) after subcloning an RT-PCR fragment made from E12.5 mouse brain RNA using the following primers:

5' Primer - 5'ACA TGA GGG TCA TTC AGG TGT GGT 3'

3' Primer - 5' TGT GCT TGG AAT CTC GAC CCT TCA 3'

## Authors' contributions

DSH performed experiments and prepared the manuscript. CB and BC performed experiments. BB contributed *Gli1/2 -/- *embryos. JDK designed experiments and prepared the manuscript. All authors read and approved the manuscript.
